# Understanding Vascular Calcification in Chronic Kidney Disease: Pathogenesis and Therapeutic Implications

**DOI:** 10.3390/ijms252313096

**Published:** 2024-12-05

**Authors:** Chiara Siracusa, Nicole Carabetta, Maria Benedetta Morano, Marzia Manica, Antonio Strangio, Jolanda Sabatino, Isabella Leo, Alberto Castagna, Eleonora Cianflone, Daniele Torella, Michele Andreucci, Maria Teresa Zicarelli, Michela Musolino, Davide Bolignano, Giuseppe Coppolino, Salvatore De Rosa

**Affiliations:** 1 Department of Medical and Surgical Sciences, “Magna Grecia” University, 88100 Catanzaro, Italy; chiara.siracusa@unicz.it (C.S.); nicole.carabetta95@gmail.com (N.C.); mariabenedetta.morano@studenti.unicz.it (M.B.M.); marzia.manica@studenti.unicz.it (M.M.); alberto.castagna@unicz.it (A.C.); cianflone@unicz.it (E.C.); mteresa.zicarelli@gmail.com (M.T.Z.); mikymusolino@gmail.com (M.M.); dbolignano@unicz.it (D.B.); saderosa@unicz.it (S.D.R.); 2 Department of Experimental and Clinical Medicine, “Magna Grecia” University, 88100 Catanzaro, Italy; antonio.strangio@unicz.it (A.S.); sabatino@unicz.it (J.S.); isabella.leo@unicz.it (I.L.); dtorella@unicz.it (D.T.); 3 Department of Health Sciences, “Magna Grecia” University, 88100 Catanzaro, Italy; andreucci@unicz.it

**Keywords:** vascular calcification, chronic kidney disease, cardiovascular risk, aortic stenosis, precision medicine

## Abstract

Vascular calcification (VC) is a biological phenomenon characterized by an accumulation of calcium and phosphate deposits within the walls of blood vessels causing the loss of elasticity of the arterial walls. VC plays a crucial role in the incidence and progression of chronic kidney disease (CKD), leading to a significant increase in cardiovascular mortality in these patients. Different conditions such as age, sex, dyslipidemia, diabetes, and hypertension are the main risk factors in patients affected by chronic kidney disease. However, VC may occur earlier and faster in these patients if it is associated with new or non-traditional risk factors such as oxidative stress, anemia, and inflammation. In chronic kidney disease, several pathophysiological processes contribute to vascular calcifications, including osteochondrogenic differentiation of vascular cells, hyperphosphatemia and hypercalcemia, and the loss of specific vascular calcification inhibitors including pyrophosphate, fetuin-A, osteoprotegerin, and matrix GLA protein. In this review we discuss the main traditional and non-traditional risk factors that can promote VC in patients with kidney disease. In addition, we provide an overview of the main pathogenetic mechanisms responsible for VC that may be crucial to identify new prevention strategies and possible new therapeutic approaches to reduce cardiovascular risk in patients with kidney disease.

## 1. Introduction

Vascular calcification (VC) is a common pathological condition in patients with chronic kidney disease (CKD), characterized by the accumulation of calcium and phosphate deposits in the walls of blood vessels [1]. This process leads to the loss of arterial elasticity, increasing the risk of cardiovascular damage. CKD patients exhibit a significantly higher risk of cardiovascular mortality compared to the general population, primarily due to their increased predisposition to vascular calcification [2].

The pathogenesis of VC in CKD patients is complex. It involves both traditional risk factors, such as hypertension, diabetes, and dyslipidemia, and non-traditional risk factors, including chronic inflammation and mineral metabolism disorders [3]. In particular, dysfunction of calcium and phosphate metabolism, typical of CKD patients, promotes mineral deposition in the arterial wall, leading to vascular stiffness and subsequently increasing blood pressure [4]. These factors contribute to the acceleration of VC by promoting the trans-differentiation of vascular smooth muscle cells (VSMCs) into an osteoblast-like phenotype, resulting in the formation of bone tissue within blood vessels [5]. The degradation of elastin, along with the loss of proteins such as matrix GLA protein (MGP) and fetuin-A, natural calcification inhibitors, further accelerates disease progression [6,7]. Given the severity of this condition, it is essential to deepen our understanding of the pathophysiological mechanisms underlying VC to develop more effective therapeutic options for managing and preventing this condition in CKD patients [1]. Currently, available therapeutic options are limited and present conflicting results. Although drugs such as phosphate binders and statins are commonly used in the management of comorbidities associated with chronic kidney disease, they show variable effects on VC progression. Moreover, certain treatments, such as vitamin K antagonists, may even worsen vascular calcifications, highlighting the need for more targeted therapeutic strategies [8].

The management of VC in CKD patients requires, therefore, a multidisciplinary approach, which not only addresses calcium and phosphate metabolism but also takes into account inflammation and other cardiovascular risk factors [9].

In this review, we will examine the main pathogenic mechanisms of VC in CKD patients, highlighting both traditional and non-traditional risk factors, as well as discussing potential therapeutic strategies to reduce cardiovascular risk in this population.

## 2. Vascular Calcification (VC) in Chronic Kidney Disease Mineral-Bone Disorder (CKD-MBD)

VC is one of the most significant complications in CKD. CKD is a global health problem with increasing prevalence, affecting 697.5 million people worldwide. The mortality rate for chronic kidney disease increased by 41.5% between 1990 and 2017 [10]. Patients with CKD may have various types of calcifications that cause different pathological conditions, contributing to the progression of kidney disease and a high risk of cardiovascular mortality [11]. Indeed, cardiovascular disease is the leading cause of morbidity and mortality in patients with end-stage renal disease undergoing hemodialysis, and vascular calcifications are one of the main associated causes [12,13]. In particular, renal dysfunction leads to the deposition of calcium and phosphate salts in the walls of blood vessels, transforming them into rigid and less elastic bone-like structures [14]. One of the most serious complications of CKD is the development of VCs. VCs can develop in the two main layers of blood vessels: the intimal layer or the medial layer. Although calcium deposition within the vessels is the main pathological process in both cases, the molecular and cellular mechanisms involved are different [15]. Calcification of the intima is a pathological condition associated with the formation of atherosclerotic plaques within the walls of arteries [16]. Atherosclerosis is prevalent in patients with chronic kidney disease, and its progression is strongly associated with the development of atheroma [17]. The deposit of lipids and inflammatory cells in the innermost layer of the vessel leads to the formation of atherosclerotic lesions, resulting in lumen obstruction, rupture, and ischemia. Inflammatory cells, such as macrophages, release pro-inflammatory cytokines that stimulate smooth muscle cells and endothelial cells to produce pro-calcifying factors. The accumulation of oxidized lipids in atherosclerotic plaques promotes calcification, probably through the induction of oxidative stress and cell death [18]. Calcification of the medial layer mainly involves muscle cells of the middle tunic and is a malignant condition linked to increased cardiovascular mortality and a higher risk of amputation in patients with type 2 diabetes mellitus and end-stage renal disease [19]. Mechanisms involved in the development of medial calcification are heterogeneous and vary with different etiologies [7]. The result of medial calcification is an increase in the rigidity of the vascular wall, resulting in increased blood pressure and a higher risk of cardiovascular mortality. In CKD, hyperphosphatemia and decreased functionality of calcification-regulating proteins such as MGP and fetuin-A contribute to the development of medial calcification [20]. Several studies have shown that elastin, a key component of the extracellular matrix of elastic arteries secreted by VSMCs, contributes to the tensile strength of blood vessels and has calcium-binding properties that may facilitate the development of medial arterial calcification [21]. The decrease in intracellular and extracellular magnesium also increases oxidative stress and vasospasms, promotes inflammation, alters endothelial function, and accelerates atherogenesis [22]. Understanding the pathogenetic mechanisms behind vascular calcifications is a crucial challenge for managing patients with CKD and developing effective intervention strategies to improve their prognosis.

## 3. Risk Factors Associated to VC in CKD

VC is more prevalent in patients with CKD compared to the general population and can develop even at a young age in this context. Once VC has developed in dialysis patients, it progresses rapidly, particularly at the coronary level [23,24,25,26]. Several risk factors associated with VC have been identified. These are typically classified into traditional risk factors, which are common for both the general and CKD population, and non-traditional risk factors (Table 1). The latter predispose CKD patients to earlier and more accelerated calcification and are thought to contribute to the additional cardiovascular risk that is not captured by the Framingham risk equation in this population [27,28,29,30].

***Traditional risk factors.*** Aging is a significant risk factor for cardiovascular disease and is closely related to VC, which represents a hallmark of vascular aging. Conditions such as renal failure, bone loss, and VC are reported both in individuals with CKD and in normal aging, suggesting that tissue dysfunction and vascular remodeling may be linked through common changes in physiology, metabolism, or cellular function induced by aging [31]. Aging is also intrinsically associated with prolonged exposure to other risk factors for the development of VC.

Being male is associated with an increased risk of VC compared to being female, probably due to the protective effects attributed to estrogenic action [32,33]. A recent study showed that males have a threefold higher risk of coronary artery calcification (CAC) compared to females, with a more diffuse distribution of calcification in men. The calcification process in women was shown to be delayed by approximately 10 years [34]. Smoking is associated with VC and a higher risk of cardiovascular disease in the general population. However, few studies have investigated this association in CKD populations [35,36,37,38]. Some studies suggest that smoking may not be a significant risk factor for VC progression in CKD patients [39,40]. However, relatively short-term assessments might not be sufficient to observe significant changes in VC. A recent study showed that current smokers with CKD had a higher prevalence of VC and an increased relative risk for VC progression. Moreover, smoking cessation for more than 10 years was associated with a significantly lower prevalence of VC compared to current smokers, regardless of smoking dose [41]. The available evidence is likely insufficient for a thorough analysis of the role of smoking in the CKD population, and future studies with larger cohorts and longer follow-up periods may help clarify this issue.

In the CKD population affected by diabetes, VCs have been reported to be more common and severe compared to non-diabetics [42], with significant prognostic implications [43]. Several studies suggest that hyperglycemia and diabetes promote the dedifferentiation of VSMCs into an osteoblast-like phenotype through various pathways. In a high-glucose environment, cultured VSMCs exhibit increased expression of osteoblast-related transcription factors, which promote matrix calcification [44]. In vivo studies have documented that arterial TNF-alpha signaling activates osteogenic expression programs, driving medial calcification during the onset of diabetes [45,46]. Hyperglycemia and diabetes also significantly enhance BMP signaling in endothelial cells in vitro and in the aortic wall in vivo. Elevated vascular BMP activity has been observed in diabetic animal models, and it has been correlated with increased expression of osteogenic markers and medial calcification [47,48]. Recent studies in animals and humans with obesity or glucose intolerance have shown that the expression of stromal proteoglycans synthesized by VSMCs is increased in adipose tissue, suggesting that hyperglycemia may promote the transformation of VSMCs into osteoblast-like cells in both animals and humans with obesity or glucose intolerance [49,50]. Additionally, hyperglycemia promotes the production of reactive oxygen species (ROS), while protein kinase C (PKC) activates the polyol pathway and stimulates the production of pro-inflammatory cytokines contributing to the development of VC and endothelial damage [51,52,53,54].

The prevalence of hypertension tends to increase as renal function declines, affecting more than 80% of patients with stage 4 to 5 chronic kidney disease (CKD) [55]. Several studies indicate that hypertension is associated with significant alterations in the extracellular matrix structure and vascular cell differentiation. Vascular tissue remodeling due to hypertension may create a favorable environment for calcium deposition within the arteries. The renin-angiotensin system is involved in VSMC apoptosis, growth, and differentiation, and may therefore play a role in the development of VC [56]. Vascular smooth muscle cell apoptosis plays a critical role in the process of vascular calcification, which is a significant contributor to cardiovascular disease. The loss of VSMCs through apoptosis leads to a reduction in the cellular environment that normally inhibits calcification, promoting an osteogenic phenotype in the remaining cells. This shift is characterized by increased expression of bone-associated markers and the release of matrix vesicles, which facilitate mineral deposition. Consequently, the disruption of VSMC homeostasis through apoptosis not only accelerates the calcification process but also contributes to the overall rigidity and dysfunction of the vascular wall [16]. In vitro studies suggest that elevated levels of phosphate and calcium are responsible for inducing VSMC apoptosis through the inactivation of Bcl-2 and activation of the Bcl-2-associated death promoter, BAD. This pro-apoptotic protein triggers the activation of caspase-3, leading to cell death [57]. Following programmed cell death, VSMCs can release apoptotic bodies, increasing intracellular calcium accumulation, which further contributes to calcification [58]. Additionally, VSMC apoptosis can result in the loss and degeneration of VSMCs in the vascular media, as well as changes in the extracellular matrix composition, stimulating the release of calcium phosphate crystals that may contribute to vascular mineralization [59]. In vitro studies have also shown that inhibiting apoptosis with caspase inhibitors significantly reduces the degree of VSMC mineralization, limiting vascular calcification [60].

In animal models, calcified arteries exhibited increased expression of bone-associated proteins and downregulation of alpha-smooth muscle actin, indicating a phenotypic shift of VSMC to an osteoblast-like state. Additionally, upregulation of angiotensin II type 1 receptors and elevated levels of angiotensin II and aldosterone were observed. Furthermore, treatment with angiotensin receptor blockers significantly inhibited arterial calcification [61,62].

Dyslipidemia is highly prevalent among CKD patients. Generally, these are secondary forms linked to altered hepatic production of apolipoproteins and impaired catabolism of triglyceride-rich lipoproteins [63,64,65]. Retrospective cross-sectional studies have shown either no correlation or a paradoxical association between low serum cholesterol levels and increased mortality in hemodialysis patients [66]. In 1990, researchers identified a U-shaped relationship between cholesterol levels and mortality, with higher overall mortality observed at lower total serum cholesterol levels [67]. A more recent study suggested that the association between low total cholesterol levels and increased mortality in dialysis patients is likely due to the cholesterol-lowering effects of systemic inflammation and malnutrition, rather than a protective role of high cholesterol levels [68]. In contrast to total cholesterol levels, the correlation between high triglyceride levels and low HDL-C concentrations with the rapid progression of VC in dialysis patients is clearer [69]. However, in a randomized trial, hemodialysis patients treated with sevelamer, a non-calcium- and non-metal-containing phosphate binder, showed an improvement in VC and a significant reduction in plasma LDL cholesterol levels. It is therefore possible that the slower rate of VC progression observed in this study may have resulted from the significant lowering of LDL levels by sevelamer [25,70].

***Non-traditional risk factors.*** Time on dialysis is associated with an increased prevalence of VC [65,71], particularly in medium-caliber arteries. Data reported in the literature indicate an estimated 15% increase in the risk of developing VC for each year spent on renal replacement therapy [72].

Advanced CKD predisposes individuals to calcium and phosphate retention, especially with high dietary intake, which can promote calcification through distinct effects on VSMCs [73,74,75,76,77,78]. One study found that a 1 mg/dL increase in serum calcium corresponds to an increase in calcification equivalent to more than five years of dialysis, while a 1 mg/dL increase in serum phosphorus corresponds to nearly 2.5 years [73]. It has been clearly demonstrated that high calcium and phosphate levels act synergistically to induce mineralization in in vitro models. In the presence of elevated phosphate concentrations, even modest increases in calcium can significantly exacerbate mineralization [79]. Additionally, high phosphate levels may influence mortality and cardiovascular risk by decreasing 1,25-dihydroxyvitamin D or increasing circulating intact parathyroid hormone (iPTH) [74,80]. iPTH has also been identified as an independent determinant of VC, inflammation, and oxidative stress [81].

Oxidative stress, characterized by an excess of free radicals, is prevalent in various conditions and is marked by increased reactive oxygen species (ROS) in the vascular wall, a typical feature of atherosclerosis [82]. In patients with CKD, oxidative stress is heightened due to the reduced effectiveness of antioxidant systems and increased pro-oxidant activity associated with aging, a high prevalence of diabetes, the uremic syndrome, and the bio-incompatibility of dialysis membranes and solutions [83]. Inflammation plays a critical role in atherosclerosis and the calcification process [84]. C-reactive protein (CRP), a well-studied inflammatory marker, has been suggested to influence vascular wall calcification [79,85], with most CKD patients exhibiting CRP levels >1.1 mg/dL [81].

The hormone fibroblast growth factor 23 (FGF23) is predominantly expressed in osteocytes and plays a crucial role in mineral homeostasis by inducing hyperphosphaturia, inhibiting calcitriol synthesis, and suppressing parathyroid hormone (PTH) secretion [86]. Several studies have demonstrated an association between FGF23 levels and VC in CKD patients [87,88]. However, the precise mechanisms by which FGF23 influences VC remain unclear.

Emerging evidence indicates that VC is influenced by complex, not yet fully elucidated, tissue-specific cellular mechanisms and circulating mediators, with osteopontin, osteoprotegerin, bone morphogenic proteins, matrix Gla protein gene, and fetuin-A being among the most frequently reported [89,90,91]. In hemodialysis patients, cathepsin-K, a cysteine protease essential for bone and extracellular matrix remodeling, has recently been identified as a crucial contributor to the development of several conditions linked to cardiovascular disease, such as atherosclerosis, obesity, diabetes, and vascular calcification [92,93]. In kidney transplant recipients, lower cathepsin-K levels indicate a time-related improvement in the uremic environment, cardiac adaptations, and, most notably, the extent of subclinical atherosclerosis. Therefore, measuring cathepsin-K in renal transplant patients could be valuable for enhancing early cardiovascular risk assessment [94].

An increasing number of studies have highlighted the importance of cathepsin K (CatK) protease in bone remodeling [95]. In mice models characterized by deficiency of cathepsin K, an osteopetrotic phenotype is observed, with ultrastructural alterations of the osteoclasts, caused by the inability of the latter to reabsorb bone tissue. The lack of cathepsin K inhibits the activity of osteoclasts, leading to the accumulation of bone matrix and development of ectopic calcifications [96]. In addition, increasing evidence suggests that cathepsin K also plays a role in early arterial calcification. It has been shown that the activity of cathepsin K precedes osteogenesis already at 20 weeks of age and is located in areas of calcification of mice with apolipoprotein deficiency E (apoE-/-) at 30 weeks.

Other strong evidence shows that calciprotein particles (CPPs), nano-sized mineralized structures, play a crucial role in the process of VC. These particles form as a result of abnormal calcium and phosphate metabolism and are particularly prevalent in CKD. CPPs serve a protective role by sequestering excess phosphate and preventing calcification, but their accumulation can contribute to vascular stiffness and dysfunction. Finally, CPPs are active players that influence smooth muscle cell differentiation and inflammatory responses within the vascular wall, triggering pathways that promote osteogenic differentiation, leading to a feedback loop that exacerbates the calcification process [97]. Some nutritional factors are considered as non-traditional risk factors for VC formation. As an example, magnesium regulates vascular smooth muscle contraction and endothelial function, and its deficiency has been linked to an increased risk of calcification. It helps inhibit vascular smooth muscle cell differentiation into osteoblast-like cells, thereby reducing the deposition of calcium in the vasculature [98]. Deficiencies in zinc have been linked with increased inflammation and vascular oxidative damage, thus promoting calcification [98]. Relevant studies show that a higher magnesium to zinc ratio is linked to increased coronary artery calcification risk, potentially due to elevated IL-6 levels [99].

## 4. Pathogenetic Mechanisms of Vascular Calcification in Chronic Kidney Disease (CKD)

**Figure 1:**
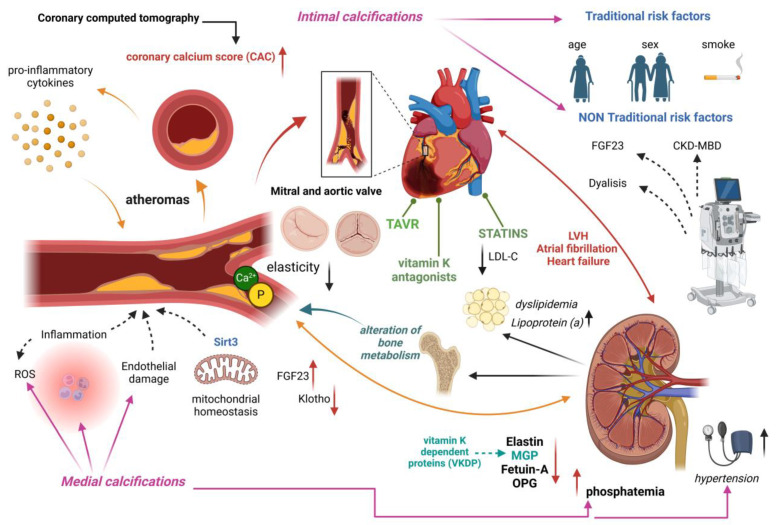
Pathogenic mechanisms of vascular calcification (VC) in chronic kidney disease (CKD). Hyperphosphatemia and hypercalcemia with loss of calcification inhibitors such as fetuin A, osteoprotegerin, and matrix GLA protein (MGP) promote osteochondrogenic differentiation of vascular cells. VC is, furthermore, influenced by traditional risk factors (e.g., aging, smoking) and non-traditional factors related to calcium-phosphorus metabolism dysfunction and increased FGF23. The accumulation of minerals in the arterial walls and the degradation of elastin lead to progressive vascular stiffness. Inflammation and the accumulation of oxidized lipids in plaques further promote calcification, negatively affecting vascular compliance and increasing the risk of cardiovascular complications (aortic and mitral valve calcifications, left ventricular hypertrophy).

VC is a critical pathological condition among patients with CKD. Despite its severe clinical implications, the molecular mechanisms underlying this process remain poorly understood [100]. A deeper understanding of these mechanisms is essential for developing targeted therapeutic strategies to mitigate the deleterious effects of vascular calcification in CKD [101] (Figure 1).

***Hyperphosphatemia and VSMCs transdifferentiation.*** Maintaining a correct balance between calcium and phosphorus is crucial, as their elevation not only induces calcification but is also closely associated with increased mortality [102]. In CKD, reduced renal capacity to eliminate phosphate leads to elevated blood levels [103]. Hyperphosphatemia and hypercalcemia stimulate VC by promoting the deposition of calcium salts in the vessel walls, particularly in the medial layer [104]. Under pro-osteogenic conditions, vascular smooth muscle cells (VSMCs) acquire an osteoblast-like phenotype, initiating the production of bone matrix within vascular walls. Calcification of the medial layer is preceded by elastin degradation mediated by esterases and metalloproteinases [105]. Elastin fragmentation is an early event in VC, and resulting elastin fragments can act as nucleation sites for hydroxyapatite formation. Osteo/chondrogenic transdifferentiation is characterized by the expression of osteogenic and chondrogenic transcription factors, including Runx2, RANK-L, alkaline phosphatase (ALP), type 2 collagen, osteocalcin, sclerostin, and low levels of magnesium [106]. Activation of the Wnt/β-catenin pathway regulates cellular differentiation and tissue homeostasis, promoting VSMC transdifferentiation and calcium deposition during CKD [107]. Moreover, elevated phosphate levels appear to increase PTH, which further stimulates calcification by overexpressing RANKL.

***Inflammation.*** Several factors contribute to VC, including inflammation and oxidative stress with increased ROS production. Patients with CKD exhibit elevated inflammatory markers such as IL-6, IL-1, and CRP [101,108]. In vitro studies suggest that TNF and oxidized low-density lipoproteins alter gene expression, inducing a phenotypic switch in VSMCs toward an osteogenic phenotype. Inflammation promotes osteogenic transdifferentiation by activating the BMPR-JANK pathway. CKD patients also show reduced levels of NRF2 and superoxide dismutase, resulting in increased NADPH oxidase activity [109]. These mechanisms compromise antioxidants, leading to increased ROS production and inflammation [110].

***Endothelial dysfunction.*** CKD patients also experience mitochondrial dysfunction, contributing to the acetylation of mitochondrial proteins. Sirt3 protein appears to regulate these acetylation levels, contributing to mitochondrial homeostasis [111]. Atherosclerosis and albuminuria are common in CKD and contribute to endothelial dysfunction by increasing von Willebrand factor levels. Additional factors include ADMA and indoxyl sulfate, which inhibit eNOS and increase FGF23, with reduced expression of Klotho. The relationship between Klotho and FGF-23 is characterized by a regulatory feedback loop; higher FGF-23 levels usually correspond to lower Klotho levels and vice versa. This interplay is crucial for maintaining phosphate and calcium balance, and disturbances in this balance can have significant pathological implications. FGF23 is crucial for phosphate and vitamin D metabolism; reduced Klotho in CKD compromises its function, contributing to hyperphosphatemia and calcification. FGF23 and increased phosphate compromise vasodilation by increasing ROS and reducing NO synthase. Further reduction in Klotho exacerbates NO synthesis impairment [112].

Klotho is considered a protective protein with antioxidant and antiapoptotic properties, and its depletion is associated with premature vascular aging in CKD patients. The loss of Klotho in aortic endothelial and smooth muscle cells even correlates with decrease in SIRT1 (sirtuin-1), which shares similar protective properties [113]. SIRT1 inhibits ROS production, while its blockade leads to a pro-inflammatory state and impaired endothelial function. Beyond combating inflammation and oxidative stress, SIRT1 may also protect against vascular calcification, positioning it as a potential therapeutic target in CKD management [114]. Last but not least, production of uremic toxins and gut microbiome alterations can play a significant role in influencing VC by disrupting normal metabolic processes and promoting inflammatory pathways [115].

***Loss of vascular calcification inhibitors.*** Among molecules acting as inhibitors of vascular mineralization, matrix Gla protein (MGP) holds significant importance as one of the most potent natural inhibitors of arterial calcification, belonging to the vitamin-K-dependent protein (VKDP) family [116]. MGP requires vitamin K as a cofactor for γ-carboxylation and post-translational phosphorylation to become biologically active. Vitamin K is involved in regulating bone metabolism, blood coagulation, and vascular health. In various patients, the inactive form of MGP has been observed, indicating a deficit in vitamin K, which influences VC development [117,118]. Among the most significant inhibitors of calcifying activity in vascular walls are fetuin-A and osteoprotegerin (OPG). Fetuin-A is well known for inhibiting osteogenesis and thereby preventing vascular calcification [119].

Fetuin-A is a glycoprotein found in serum and primarily synthesized by the liver. It experiences significant post-translational modifications that enhance its biological function and interacts with calcium and phosphate, preventing their accumulation within the vascular system [120,121]. On the other hand, OPG acts by inhibiting osteoclast differentiation, regulating bone resorption through its interaction with RANKL receptor [122]. Increased reduction of these inhibitors promotes microcalcification formation in vascular walls, thereby increasing VC and the risk of cardiovascular complications in CKD patients.

## 5. Clinical Impact and Management Strategies of CV in CKD

The onset of cardiovascular disease (CVD) is common in patients with CKD. Individuals with CKD have a significant increased risk of cardiovascular events: 50% of patients with stage 4/5 CKD develop CVD. Moreover, cardiovascular mortality in advanced forms of renal failure accounts for about 40–50% of all deaths [123]. Coronary artery disease (CAD) is linked to both traditional and non-traditional CVD risk factors, as well as dialysis-related factors; additionally, vascular calcifications (VC) rise with decreasing glomerular filtration rate (GFR) and are linked to mortality in end-stage kidney disease [124,125,126].

**Figure 2:**
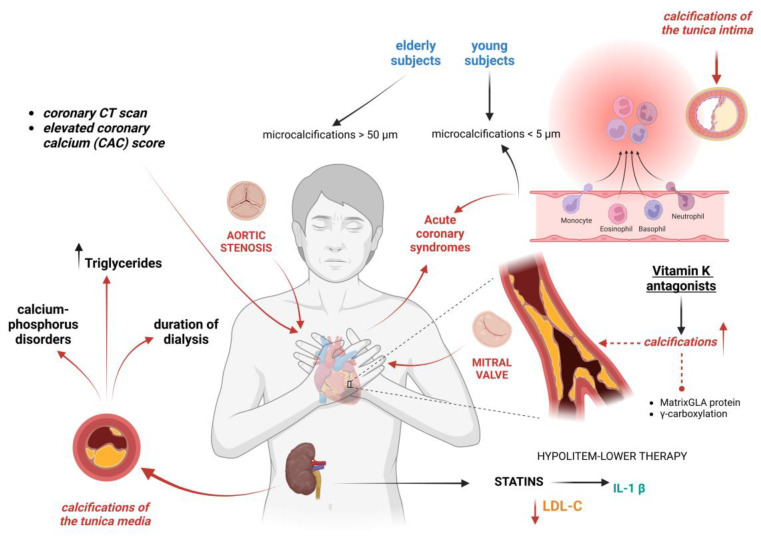
Clinical management of vascular calcifications in CKD patients and intervention strategies to delay the progression.

Patients may have varying degrees of calcification in different tissues, and this can have an impact on cardiovascular outcomes. As a result, calcification-targeting therapy strategies will probably need customized regimens depending on each patient’s characteristics (Figure 2). According to autopsy investigations, CKD patients have more advanced atherosclerotic plaques and calcified atherosclerotic lesions compared to patients without CKD [127,128].

**Figure 3:**
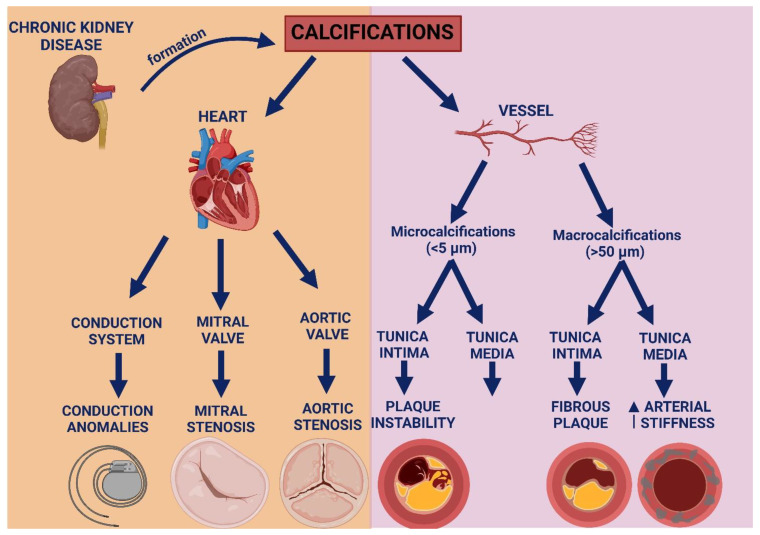
Role of micro- and macro-calcifications in the large vessels, heart valves, and cardiac conduction system.

Calcifications of coronary arteries, abdominal aorta, aortic valve, and superficial femoral arteries are prevalent in patients with end-stage CKD [129,130,131,132,133,134,135]. Medial artery calcification, once thought to be a benign feature and unrelated to stenosis, it is now recognized as a malignant feature, associated with an increased cardiovascular mortality. Medial calcification appears distributed along elastic fibers, promoting an increased arterial stiffness, rather than atherosclerosis. Furthermore, it involves more peripheral arteries, with an increased risk of limb ischemia and adverse outcomes [136,137,138]. Medial calcification, in patients without traditional atherosclerotic risk factors, is strongly associated with high blood triglycerides, calcium-phosphate disorders, and duration of dialysis. This may be due to a specific response to kidney disease, as in younger subjects there is rarely calcification of the tunica media [139,140]. VC can also accumulate in the tunica intima of the vessel, promoting vasal remodeling and accelerated plaque formation, with collagen production and chronic inflammation. Traditionally, a low collagen content in the fibrous cap is linked to plaque vulnerability [141]. Younger subjects are more likely to experience microcalcifications (<5 µm) in the tunica intima, linked to inflammation and plaque instability that may result in acute coronary syndromes (ACS), while elderly individuals with multivessel and stable CAD are more likely to develop macrocalcifications (>50 µm) in the tunica intima that decrease plaque vulnerability and promote plaque stabilization (Figure 3). However, although large calcifications can stabilize plaques temporarily, they can also cause vessel stenosis and consequently reduced blood flow and elevated systemic resistance [142]. The presence of microcalcifications at the coronary CT is a high-risk criterion of instability [143]. At the cardiac CT, an elevated coronary artery calcium score (CAC) is correlated with the severity of CAD and increased cardiovascular morbidity [144,145,146,147]. In fact, calcified coronary lesions present specific challenges, are characterized by higher complication rates, and require a dedicated therapeutic approach [148].

In more complex scenarios of extensive coronary artery disease, chronic total occlusions (CTOs), patients with chronic kidney disease have a higher likelihood of presenting heavy calcification patterns, which significantly increase procedural complexities [149]. New methods to detect coronary calcification are under development, which might further simplify the assessment of calcification and the integration of this information in the clinical management of patients [150]. Lipid lowering is the therapeutic cornerstone in these patients. Abnormal levels of lipids are frequently observed in patients with CKD; therefore, since CKD is a risk factor, an aggressive LDL-C lowering is justified if renal function is impaired [151]. An LDL-C reduction was shown to substantially decrease cardiovascular risk and all-cause mortality in the CKD population [152]. However, the cardiovascular benefit of lowering LDL-C decreases at the same time as reduced kidney function; this is possible as the presence of other risk factors contribute to this condition, such as inflammation and oxidative stress [153,154]. Statins have advantages that extend beyond their ability to decrease cholesterol; in fact, they promote the release of IL-1 beta by macrophages, an inflammatory cytokine that stimulates vascular muscle cells to take on an osteogenic phenotype. This results in greater plaque stability [155,156,157]. Hence, the management of dyslipidemia in chronic kidney disease (CKD) can be particularly challenging. In fact, the characterized profile associated with CKD of elevated triglycerides, reduced HDL, and a shift towards small, dense LDL particles, which are more atherogenic, leads to an even higher risk profile. In addition, lipoprotein(a) is often elevated, and lipoprotein catabolism is impaired due to reduced enzyme activity and receptor function. CKD-associated inflammation and oxidative stress lead to further modification of lipoproteins, increasing cardiovascular risk. Uremia and dialysis, particularly peritoneal dialysis, exacerbate lipid abnormalities. Medications used in CKD management and conditions like nephrotic syndrome can also worsen dyslipidemia. Despite this, statins may have reduced efficacy in advanced CKD, particularly in dialysis patients, necessitating comprehensive management of both lipid and non-lipid risk factors [158,159].

Calcifications can also accumulate in the heart valves, causing increased valve stiffness and altered hemodynamics. Calcific aortic valve disease causes aortic stenosis with systemic flow impairment and an increased cardiac afterload [160]. In a similar way, calcifications can accumulate on the mitral valve causing flow obstruction and increased filling pressures [161]. In fact, patients with aortic stenosis in the presence of a bicuspid aortic valve present a characteristic modulation of the epigenetic pathways regulating calcification [162]. Transcatheter aortic valve replacement (TAVR) has improved outcomes in patients with severe aortic stenosis, although the asymmetric distribution of calcifications could make the procedure more complicated [163] (Figure 3).

In rat models, several drugs used to treat mineral disturbances have protective effects; nevertheless, no medication has been proven to reliably lower cardiovascular calcifications and improve cardiovascular outcomes in CKD patients [8]. Furthermore, some drugs such as vitamin K antagonists used in patients with end-stage CKD and atrial fibrillation appear to be involved in VC, through mechanisms that include matrix Gla protein (MGP) inhibition and the γ-carboxylation of other proteins that regulate mineralization [7].

However, some of the medications used in the treatment of secondary hyperparathyroidism and hyperphosphatemia are far from beneficial, as they promote the formation of vascular calcifications. For example, certain calcium-based phosphate binders, such as calcium carbonate, or even the supplementation of calcitriol, encourage the presence of free calcium ions in the blood, which tend to precipitate in the walls of blood vessels and promote calcifications [164,165]. Thus, while the intent to regulate calcium-phosphorus metabolism is made possible through calcium and vitamin D supplementation, the side effects related to vascular calcifications promoted by these medications should also be considered. In a recent study involving stage 5 CKD patients, those using calcium-based phosphate binders showed a higher dose-dependent risk of coronary heart disease and CV compared to nonusers [166]. Other trials have linked the use of calcitriol with cardiovascular abnormalities including intima-media thickness and coronary artery calcification, even in patients affected by CKD since childhood [167]. Moreover, some studies performed in rat models with CKD revealed that calcitriol administration at doses as low as 20 ng/kg can exacerbate the severity of vascular calcification, independent of vitamin K levels [168]. On the other hand, some other drugs have been revealed to be protective in this sense. It was demonstrated that in hemodialysis patients, calcimimetics not only address excessive PTH secretion but also reduce vascular calcification, influencing the activity of the vascular calcium sensor [169].

## 6. Platelet Aggregation and Adhesion in Vascular Calcifications

Although platelet dysfunction in CKD is established, the information regarding the type of dysfunction is not entirely consistent. CKD has been linked to endothelial cell activation and elevated levels of von Willebrand factor. Furthermore there is an imbalance between platelet modulators such as adenosine diphosphate (ADP), serotonin, and cyclic adenosine monophosphate (Camp); a decrease in thromboxane A2 production; and an increase in intracellular calcium [170,171]. Lastly, there is a decreased bioavailability of nitric oxide, a powerful dilatator and inhibitor of platelet adhesion and aggregation [172,173,174]. There are many pathophysiological factors that can cause a dysregulation of platelets, including uremia toxins and inflammatory cytokines, which can promote the formation of platelet microvesicles and the production of thrombin. These factors can mediate platelet-monocyte aggregation and be directly absorbed by VSMc, which further contributes to VC in patients with CKD [175]. The interaction between VSMc and inflammatory cytokines causes a phenotypic switch of VSMc, resulting in an osteogenic phenotype that facilitates the formation of VC [176].

Platelets also express osteocalcin, a protein produced primarily by the osteoblasts during bone formation, which seems to be directly related to the formation of VC [177,178]. In addition, platelets can secrete numerous chemokines that promote VC and atherosclerosis, including chemokine ligand 5 (CCL5), chemokine ligand 4 (CXCL4), chemokine ligand 12 (CXCL12), and chemokine ligand 16 (CXCL16), which may induce a local inflammatory state, with recruitment of leukocyte. These mechanisms improve the interaction between leukocytes and endothelial cells, thereby indirectly enhancing atherosclerosis and vascular fibrosis [179,180,181].

In summary, the increased aggregation and platelet adhesion in CKD, due to increased production of proinflammatory factors and an imbalance of platelet modulators, promote fibrosis, VC, and atherosclerosis.

## 7. Lifelong Risk of CKD: Impact of Heterotopic Calcification

VC refers to a process that leads to a pathological accumulation of calcium in the intima and media layers of the vessel wall [179]. Intimal calcifications are associated with classic atherogenic risk factors, such as ageing, dyslipidemia, diabetes, hypertension, and cigarette smoking, and are associated with the development of atherosclerotic plaque [182]. Although not specifically linked with chronic kidney disease (CKD), the decline of renal function may act as an accelerator of the atherosclerotic process [183]. Medial calcification appears instead to be associated with calcium-phosphate levels, altered mineral metabolism, and CKD stage; it tends to be more diffuse and located in regions usually spared from atherosclerosis [184]. Medial calcification generally appears uniformly distributed along elastic fibers and does not generally coexist with the lipid and inflammatory remodeling mechanisms observed in atherosclerosis [182]. This pathological mechanism induces arterial stiffening and increase the risk of fibrosis, inflammation, and oxidative stress [101]. VC has a greater incidence among patients with CKD if compared to non-CKD patients [185]. CKD patients with confirmed medial vascular calcification have significantly higher rates of all-cause and cardiovascular mortality compared patients without medial calcification [124]. A reduction in vascular compliance determines higher systolic blood pressure, which increases cardiac work, leading in turn to left ventricular hypertrophy, a common finding in the advanced stages of CKD (3 or more). This also provides the basis for the augmented rates of heart failure and atrial fibrillation observed in these groups of patients [186]. There is also a correlation between renal disease and coronary artery disease; in particular, CKD patients present greater coronary artery calcium (CAC) score values than controls [187], with the mean value of the score related to the grade of renal impairment [188]. A large prospective cohort study showed that, among patients with impaired renal function, being 1 standard-deviation log higher in CAC score was significantly associated with a 40% higher risk of cardiovascular disease, a 44% higher risk of myocardial infarction, and a 39% higher risk of heart failure after adjusting for risk factors [189]. Another study involving 133 patients with type 2 diabetes mellitus found that medial VCs were an independent and powerful predictor of cardiovascular mortality, with a stronger impact than intimal VCs. Medial calcifications can also be detected in peripheral arteries, where they are related to limb ischemia, foot ulcers, increased risk of amputation, and other adverse outcomes [136,138]. CKD associates with the calcification of cardiac valves, in particular the aortic valve leaflets and mitral valve anulus [190]. Valve calcification is associated with a significantly increased risk for all-cause mortality in hemodialysis patients, especially if calcification of both valves is present [191]. A great percentage of hemodialysis patients presents calcification of the abdominal aorta [134], the severity of which has been shown to be a strong predictor of adverse outcomes, including all-cause mortality and both fatal and non-fatal cardiovascular events [192]. Measuring breast arterial calcification gives indirect information about medial calcification, because these vessels are not generally affected by atherosclerosis [193], and recent findings suggest that it is strongly and independently associated with peripheral artery disease in women with end-stage CKD and may be predictive of adverse clinical events [194].

## 8. Diagnosis and Management of VCs in CKD

Different clinical studies have sought to better understand the above-mentioned risk factors for vascular calcifications and translate findings into better diagnostic tools and management strategies for VC in CKD patients. Techniques such as computed tomography (CT) scanning and ultrasound are often used to quantify VC, with CT being the most sensitive technique for evaluation of VC [195]. Recent studies have focused on identifying specific imaging biomarkers that correlate with overall cardiovascular risk. Various biomarkers are under investigation, including serum levels of osteocalcin, Klotho, and FGF23, which may help identify patients at higher risk of developing VC [112]. Various trials assessing the effects of phosphate binders, vitamin D analogs, and calcimimetics have been conducted to see if they can slow the progression of VC in patients with CKD [196]. Other drugs such as calcimimetic agents increase the sensitivity of CaSR to extracellular calcium and decrease PTH secretion [197]. Calcitriol is associated with hypercalcemia and hyperphosphatemia, so some patients may not benefit from its use. Moreover, parathyroidectomy could be taken into consideration for several conditions, such as hyperparathyroidism that does not respond to calcimimetics, hyperparathyroidism in dialysis patients who resists medical therapy, refractory hyperphosphatemia, and calciphylaxis [198]. Moreover, phosphate binders could help since they form a non-absorbable complex in the gastrointestinal tract, preventing phosphate absorption [199]. Finally, tenapanor, an experimental phosphate absorption inhibitor, blocks the intestinal sodium/hydrogen exchanger isoform 3 [200]. Addressing traditional risk factors through lifestyle changes, such as diet and smoking cessation, is integral to managing VC [201]. As an example, the diet of patients affected by CKD should not include processed food, because food additives contain inorganic highly absorbable phosphate, and dietary phosphate should be less than 800 mg daily in patients with CKD under dialysis. Dietary calcium intake should be around 1000 mg/day, and calcium-containing drugs should be avoided [202]. 

Ongoing research into agents targeting specific pathways involved in VC may provide new options for managing this complication in CKD.

## 9. Conclusions

Vascular calcification in chronic kidney disease is a multifaceted condition influenced by a mix of traditional and novel risk factors, making it a critical area of research with significant implications for patient outcomes. An enhanced understanding of these risk factors through clinical studies can inform improved diagnostic approaches and management strategies, ultimately reducing the cardiovascular risk associated with CKD. Future advancements in this field are expected to focus on innovative imaging techniques and biomarkers that allow for the early diagnosis of VC, enabling more accurate assessments of calcification processes. Additionally, there is a growing emphasis on exploring novel pharmacological agents and dietary interventions that may help mitigate the progression of calcification while addressing the mineral and bone disorders commonly seen in CKD. Integrating insights from basic research into the mechanisms underlying VC, such as the roles of inflammation, oxidative stress, and various signaling pathways, could pave the way for developing targeted therapies. Future research should continue to investigate potential therapeutic interventions that effectively address VC in the context of CKD.

## Figures and Tables

**Table 1:** Risk factors for vascular calcification.

Traditional Risk Factors	Non-Traditional Risk Factors
Ageing	Dialysis vintage
Sex	Disordered mineral metabolism: calcium, phosphate
Smoking	Oxidative stress and inflammation
Diabetes	Others: FGF-23 soluble Klotho, osteopontin, osteoprotegerin, bone morphogenic proteins, matrix Gla protein gene, fetuin-A, calciprotein particles (CPP), magnesium, zinc, microbiome, uremic toxin, and advanced glycation end-products
Hypertension	
Dyslipidemia	

## References

[1] Jankowski J., Floege J., Fliser D., Böhm M., Marx N. (2021). Cardiovascular Disease in Chronic Kidney Disease: Pathophysiological Insights and Therapeutic Options. Circulation.

[2] Wang P.-W., Zhang A.-H. (2022). New mechanisms of chronic kidney disease-associated vascular calcification. Sheng Li Xue Bao.

[3] Liabeuf S., Desjardins L., Diouf M., Temmar M., Renard C., Choukroun G., Massy Z.A. (2015). The Addition of Vascular Calcification Scores to Traditional Risk Factors Improves Cardiovascular Risk Assessment in Patients with Chronic Kidney Disease. PLoS ONE.

[4] Jono S., McKee M.D., Murry C.E., Shioi A., Nishizawa Y., Mori K., Morii H., Giachelli C.M. (2000). Phosphate regulation of vascular smooth muscle cell calcification. Circ. Res..

[5] Zhang Y.-X., Tang R.-N., Wang L.-T., Liu B.-C. (2021). Role of crosstalk between endothelial cells and smooth muscle cells in vascular calcification in chronic kidney disease. Cell Prolif..

[6] Roumeliotis S., Dounousi E., Salmas M., Eleftheriadis T., Liakopoulos V. (2020). Vascular Calcification in Chronic Kidney Disease: The Role of Vitamin K- Dependent Matrix Gla Protein. Front. Med..

[7] Siracusa C., Carino A., Carabetta N., Manica M., Sabatino J., Cianflone E., Leo I., Strangio A., Torella D., De Rosa S. (2024). Mechanisms of Cardiovascular Calcification and Experimental Models: Impact of Vitamin K Antagonists. J. Clin. Med..

[8] Himmelsbach A., Ciliox C., Goettsch C. (2020). Cardiovascular Calcification in Chronic Kidney Disease-Therapeutic Opportunities. Toxins.

[9] Kanbay M., Copur S., Tanriover C., Yavuz F., Galassi A., Ciceri P., Cozzolino M. (2023). The pathophysiology and management of vascular calcification in chronic kidney disease patients. Expert. Rev. Cardiovasc. Ther..

[10] Carney E.F. (2020). The impact of chronic kidney disease on global health. Nat. Rev. Nephrol..

[11] Hénaut L., Chillon J.-M., Kamel S., Massy Z.A. (2018). Updates on the Mechanisms and the Care of Cardiovascular Calcification in Chronic Kidney Disease. Semin. Nephrol..

[12] Fox C.S., Matsushita K., Woodward M., Bilo H.J.G., Chalmers J., Heerspink H.J.L., Lee B.J., Perkins R.M., Rossing P., Sairenchi T. (2012). Associations of kidney disease measures with mortality and end-stage renal disease in individuals with and without diabetes: A meta-analysis. Lancet.

[13] Mahmoodi B.K., Matsushita K., Woodward M., Blankestijn P.J., Cirillo M., Ohkubo T., Rossing P., Sarnak M.J., Stengel B., Yamagishi K. (2012). Associations of kidney disease measures with mortality and end-stage renal disease in individuals with and without hypertension: A meta-analysis. Lancet.

[14] Kim J.S., Hwang H.S. (2021). Vascular Calcification in Chronic Kidney Disease: Distinct Features of Pathogenesis and Clinical Implication. Korean Circ. J..

[15] Hutcheson J.D., Goettsch C. (2023). Cardiovascular Calcification Heterogeneity in Chronic Kidney Disease. Circ. Res..

[16] Durham A.L., Speer M.Y., Scatena M., Giachelli C.M., Shanahan C.M. (2018). Role of smooth muscle cells in vascular calcification: Implications in atherosclerosis and arterial stiffness. Cardiovasc. Res..

[17] Caffarelli C., Montagnani A., Nuti R., Gonnelli S. (2017). Bisphosphonates, atherosclerosis and vascular calcification: Update and systematic review of clinical studies. Clin. Interv. Aging.

[18] Vervloet M., Cozzolino M. (2017). Vascular calcification in chronic kidney disease: Different bricks in the wall?. Kidney Int..

[19] Pongsuwan K., Kusirisin P., Narongkiattikhun P., Chattipakorn S.C., Chattipakorn N. (2022). Mitochondria and vascular calcification in chronic kidney disease: Lessons learned from the past to improve future therapy. J. Cell Physiol..

[20] Giachelli C.M. (2009). The emerging role of phosphate in vascular calcification. Kidney Int..

[21] Toussaint N.D. (2011). Extracellular matrix calcification in chronic kidney disease. Curr. Opin. Nephrol. Hypertens..

[22] Tangvoraphonkchai K., Davenport A. (2018). Magnesium and Cardiovascular Disease. Adv. Chronic Kidney Dis..

[23] Goodman W.G., Goldin J., Kuizon B.D., Yoon C., Gales B., Sider D., Wang Y., Chung J., Emerick A., Greaser L. (2000). Coronary-artery calcification in young adults with end-stage renal disease who are undergoing dialysis. N. Engl. J. Med..

[24] Oh J., Wunsch R., Turzer M., Bahner M., Raggi P., Querfeld U., Mehls O., Schaefer F. (2002). Advanced coronary and carotid arteriopathy in young adults with childhood-onset chronic renal failure. Circulation.

[25] Chertow G.M., Burke S.K., Raggi P., Treat to Goal Working G. (2002). Sevelamer attenuates the progression of coronary and aortic calcification in hemodialysis patients. Kidney Int..

[26] Moe S.M., O’Neill K.D., Fineberg N., Persohn S., Ahmed S., Garrett P., Meyer C.A. (2003). Assessment of vascular calcification in ESRD patients using spiral CT. Nephrol. Dial. Transplant..

[27] Longenecker J.C., Coresh J., Powe N.R., Levey A.S., Fink N.E., Martin A., Klag M.J. (2002). Traditional cardiovascular disease risk factors in dialysis patients compared with the general population: The CHOICE Study. J. Am. Soc. Nephrol..

[28] Cheung A.K., Sarnak M.J., Yan G., Dwyer J.T., Heyka R.J., Rocco M.V., Teehan B.P., Levey A.S. (2000). Atherosclerotic cardiovascular disease risks in chronic hemodialysis patients. Kidney Int..

[29] Sarnak M.J., Coronado B.E., Greene T., Wang S.R., Kusek J.W., Beck G.J., Levey A.S. (2002). Cardiovascular disease risk factors in chronic renal insufficiency. Clin. Nephrol..

[30] Carullo N., Andreucci M., Coppolino G. (2023). Holistic Vision of Cardiovascular Complications in Chronic Kidney Disease. Rev. Cardiovasc. Med..

[31] London G.M. (2012). Bone-vascular cross-talk. J. Nephrol..

[32] Petrović M., Brković V., Baralić M., Marić I., Petković N., Stanković S., Lalić N., Stanisavljević D., Đukanović L., Ležaić V. (2024). Comparative Analysis of Vascular Calcification Risk Factors in Pre-Hemodialysis and Prevalent Hemodialysis Adult Patients: Insights into Calcification Biomarker Associations and Implications for Intervention Strategies in Chronic Kidney Disease. Diagnostics.

[33] Tao X.Y., Zuo A.Z., Wang J.Q., Tao F.B. (2016). Effect of primary ovarian insufficiency and early natural menopause on mortality: A meta-analysis. Climacteric.

[34] Kim B.S., Chan N., Hsu G., Makaryus A.N., Chopra M., Cohen S.L., Makaryus J.N. (2021). Sex Differences in Coronary Arterial Calcification in Symptomatic Patients. Am. J. Cardiol..

[35] Liebman S.E., Lamontagne S.P., Huang L.-S., Messing S., Bushinsky D.A. (2011). Smoking in dialysis patients: A systematic review and meta-analysis of mortality and cardiovascular morbidity. Am. J. Kidney Dis..

[36] Mc Causland F.R., Brunelli S.M., Waikar S.S. (2012). Association of smoking with cardiovascular and infection-related morbidity and mortality in chronic hemodialysis. Clin. J. Am. Soc. Nephrol..

[37] Foley R.N., Herzog C.A., Collins A.J. (2003). Smoking and cardiovascular outcomes in dialysis patients: The United States Renal Data System Wave 2 study. Kidney Int..

[38] Provenzano M., Serra R., Michael A., Bolignano D., Coppolino G., Ielapi N., Serraino G.F., Mastroroberto P., Locatelli F., De Nicola L. (2021). Smoking habit as a risk amplifier in chronic kidney disease patients. Sci. Rep..

[39] Kestenbaum B.R., Adeney K.L., de Boer I.H., Ix J.H., Shlipak M.G., Siscovick D.S. (2009). Incidence and progression of coronary calcification in chronic kidney disease: The Multi-Ethnic Study of Atherosclerosis. Kidney Int..

[40] Bundy J.D., Chen J., Yang W., Budoff M., Go A.S., Grunwald J.E., Kallem R.R., Post W.S., Reilly M.P., Ricardo A.C. (2018). Risk factors for progression of coronary artery calcification in patients with chronic kidney disease: The CRIC study. Atherosclerosis.

[41] Lee M.J., Park J.T., Chang T.I., Joo Y.S., Yoo T.H., Park S.K., Chung W., Kim Y.S., Kim S.W., Oh K.H. (2021). Smoking Cessation and Coronary Artery Calcification in CKD. Clin. J. Am. Soc. Nephrol..

[42] Ishimura E., Okuno S., Taniwaki H., Kizu A., Tsuchida T., Shioi A., Shoji T., Tabata T., Inaba M., Nishizawa Y. (2008). Different risk factors for vascular calcification in end-stage renal disease between diabetics and nondiabetics: The respective importance of glycemic and phosphate control. Kidney Blood Press. Res..

[43] Lehto S., Niskanen L., Suhonen M., Rönnemaa T., Laakso M. (1996). Medial artery calcification. A neglected harbinger of cardiovascular complications in non-insulin-dependent diabetes mellitus. Arterioscler. Thromb. Vasc. Biol..

[44] Chen N.X., Duan D., O’Neill K.D., Moe S.M. (2006). High glucose increases the expression of Cbfa1 and BMP-2 and enhances the calcification of vascular smooth muscle cells. Nephrol. Dial. Transplant..

[45] Schreyer S.A., Vick C., Lystig T.C., Mystkowski P., LeBoeuf R.C. (2002). LDL receptor but not apolipoprotein E deficiency increases diet-induced obesity and diabetes in mice. Am. J. Physiol. Endocrinol. Metab..

[46] Al-Aly Z., Shao J.-S., Lai C.-F., Huang E., Cai J., Behrmann A., Cheng S.-L., Towler D.A. (2007). Aortic Msx2-Wnt calcification cascade is regulated by TNF-alpha-dependent signals in diabetic Ldlr-/- mice. Arterioscler. Thromb. Vasc. Biol..

[47] Nett P.C., Ortmann J., Celeiro J., Haas E., Hofmann-Lehmann R., Tornillo L., Terraciano L.M., Barton M. (2006). Transcriptional regulation of vascular bone morphogenetic protein by endothelin receptors in early autoimmune diabetes mellitus. Life Sci..

[48] San Martín A., Du P., Dikalova A., Lassègue B., Aleman M., Góngora M.C., Brown K., Joseph G., Harrison D.G., Taylor W.R. (2007). Reactive oxygen species-selective regulation of aortic inflammatory gene expression in Type 2 diabetes. Am. J. Physiol. Heart Circ. Physiol..

[49] Bolton K., Segal D., McMillan J., Jowett J., Heilbronn L., Abberton K., Zimmet P., Chisholm D., Collier G., Walder K. (2008). Decorin is a secreted protein associated with obesity and type 2 diabetes. Int. J. Obes..

[50] Zhang J., Wright W., Bernlohr D.A., Cushman S.W., Chen X. (2007). Alterations of the classic pathway of complement in adipose tissue of obesity and insulin resistance. Am. J. Physiol. Endocrinol. Metab..

[51] Baxevanis A.D., Bryant S.H., Landsman D. (1995). Homology model building of the HMG-1 box structural domain. Nucleic Acids Res..

[52] Sturiale A., Coppolino G., Loddo S., Criseo M., Campo S., Crasci E., Bolignano D., Nostro L., Teti D., Buemi M. (2007). Effects of haemodialysis on circulating endothelial progenitor cell count. Blood Purif..

[53] Allegra A., Coppolino G., Bolignano D., Giacobbe M.S., Alonci A., D’Angelo A., Bellomo G., Teti D., Loddo S., Musolino C. (2009). Endothelial progenitor cells: Pathogenetic role and therapeutic perspectives. J. Nephrol..

[54] Coppolino G., Bolignano D., Campo S., Loddo S., Teti D., Buemi M. (2008). Circulating progenitor cells after cold pressor test in hypertensive and uremic patients. Hypertens. Res..

[55] Centers for Disease Control and Prevention (2007). Prevalence of chronic kidney disease and associated risk factors--United States, 1999–2004. MMWR Morb. Mortal. Wkly. Rep..

[56] Savoia C., Burger D., Nishigaki N., Montezano A., Touyz R.M. (2011). Angiotensin II and the vascular phenotype in hypertension. Expert. Rev. Mol. Med..

[57] Paloian N.J., Giachelli C.M. (2014). A current understanding of vascular calcification in CKD. Am. J. Physiol. Ren. Physiol..

[58] Kapustin A.N., Davies J.D., Reynolds J.L., McNair R., Jones G.T., Sidibe A., Schurgers L.J., Skepper J.N., Proudfoot D., Mayr M. (2011). Calcium regulates key components of vascular smooth muscle cell-derived matrix vesicles to enhance mineralization. Circ. Res..

[59] Clarke M.C., Littlewood T.D., Figg N., Maguire J.J., Davenport A.P., Goddard M., Bennett M.R. (2008). Chronic apoptosis of vascular smooth muscle cells accelerates atherosclerosis and promotes calcification and medial degeneration. Circ. Res..

[60] Proudfoot D., Skepper J.N., Hegyi L., Bennett M.R., Shanahan C.M., Weissberg P.L. (2000). Apoptosis regulates human vascular calcification in vitro: Evidence for initiation of vascular calcification by apoptotic bodies. Circ. Res..

[61] Armstrong Z.B., Boughner D.R., Drangova M., Rogers K.A. (2011). Angiotensin II type 1 receptor blocker inhibits arterial calcification in a pre-clinical model. Cardiovasc. Res..

[62] Wu S.-Y., Yu Y.-R., Cai Y., Jia L.-X., Wang X., Xiao C.-S., Tang C.-S., Qi Y.-F. (2012). Endogenous aldosterone is involved in vascular calcification in rat. Exp. Biol. Med..

[63] Kronenberg F. (2005). Dyslipidemia and nephrotic syndrome: Recent advances. J. Ren. Nutr..

[64] de Mendoza S.G., Kashyap M.L., Chen C.Y., Lutmer R.F. (1976). High density lipoproteinuria in nephrotic syndrome. Metabolism.

[65] Wanner C., Quaschning T. (2001). Dyslipidemia and renal disease: Pathogenesis and clinical consequences. Curr. Opin. Nephrol. Hypertens..

[66] Kasiske B., Cosio F.G., Beto J., Bolton K., Chavers B.M., Grimm R., Levin A., Masri B., Parekh R., Wanner C. (2004). Clinical practice guidelines for managing dyslipidemias in kidney transplant patients: A report from the Managing Dyslipidemias in Chronic Kidney Disease Work Group of the National Kidney Foundation Kidney Disease Outcomes Quality Initiative. Am. J. Transplant..

[67] Lowrie E.G., Lew N.L. (1990). Death risk in hemodialysis patients: The predictive value of commonly measured variables and an evaluation of death rate differences between facilities. Am. J. Kidney Dis..

[68] Liu Y., Coresh J., Eustace J.A., Longenecker J.C., Jaar B., Fink N.E., Tracy R.P., Powe N.R., Klag M.J. (2004). Association between cholesterol level and mortality in dialysis patients: Role of inflammation and malnutrition. JAMA.

[69] Tamashiro M., Iseki K., Sunagawa O., Inoue T., Higa S., Afuso H., Fukiyama K. (2001). Significant association between the progression of coronary artery calcification and dyslipidemia in patients on chronic hemodialysis. Am. J. Kidney Dis..

[70] Coppolino G., Lucisano S., Rivoli L., Fuiano G., Villari A., Villari I., Leonello G., Lacquaniti A., Santoro D., Buemi M. (2015). Sevalamer Hydrochloride, Sevelamer Carbonate and Lanthanum Carbonate: In Vitro and In Vivo Effects on Gastric Environment. Ther. Apher. Dial..

[71] McCullough P.A., Sandberg K.R., Dumler F., Yanez J.E. (2004). Determinants of coronary vascular calcification in patients with chronic kidney disease and end-stage renal disease: A systematic review. J. Nephrol..

[72] Yuen D., Pierratos A., Richardson R.M., Chan C.T. (2006). The natural history of coronary calcification progression in a cohort of nocturnal haemodialysis patients. Nephrol. Dial. Transplant..

[73] Raggi P., Boulay A., Chasan-Taber S., Amin N., Dillon M., Burke S.K., Chertow G.M. (2002). Cardiac calcification in adult hemodialysis patients. A link between end-stage renal disease and cardiovascular disease?. J. Am. Coll. Cardiol..

[74] Benet-Pagès A., Orlik P., Strom T.M., Lorenz-Depiereux B. (2005). An FGF23 missense mutation causes familial tumoral calcinosis with hyperphosphatemia. Hum. Mol. Genet..

[75] Nasrallah M.M., El-Shehaby A.R., Salem M.M., Osman N.A., El Sheikh E., Sharaf El Din U.A.A. (2010). Fibroblast growth factor-23 (FGF-23) is independently correlated to aortic calcification in haemodialysis patients. Nephrol. Dial. Transplant..

[76] Jean G., Bresson E., Terrat J.C., Vanel T., Hurot J.M., Lorriaux C., Mayor B., Chazot C. (2009). Peripheral vascular calcification in long-haemodialysis patients: Associated factors and survival consequences. Nephrol. Dial. Transplant..

[77] Tentori F., Blayney M.J., Albert J.M., Gillespie B.W., Kerr P.G., Bommer J., Young E.W., Akizawa T., Akiba T., Pisoni R.L. (2008). Mortality risk for dialysis patients with different levels of serum calcium, phosphorus, and PTH: The Dialysis Outcomes and Practice Patterns Study (DOPPS). Am. J. Kidney Dis..

[78] Adeney K.L., Siscovick D.S., Ix J.H., Seliger S.L., Shlipak M.G., Jenny N.S., Kestenbaum B.R. (2009). Association of serum phosphate with vascular and valvular calcification in moderate CKD. J. Am. Soc. Nephrol..

[79] Reynolds J.L., Joannides A.J., Skepper J.N., McNair R., Schurgers L.J., Proudfoot D., Jahnen-Dechent W., Weissberg P.L., Shanahan C.M. (2004). Human vascular smooth muscle cells undergo vesicle-mediated calcification in response to changes in extracellular calcium and phosphate concentrations: A potential mechanism for accelerated vascular calcification in ESRD. J. Am. Soc. Nephrol..

[80] Kestenbaum B., Sampson J.N., Rudser K.D., Patterson D.J., Seliger S.L., Young B., Sherrard D.J., Andress D.L. (2005). Serum phosphate levels and mortality risk among people with chronic kidney disease. J. Am. Soc. Nephrol..

[81] Yao Q., Pecoits-Filho R., Lindholm B., Stenvinkel P. (2004). Traditional and non-traditional risk factors as contributors to atherosclerotic cardiovascular disease in end-stage renal disease. Scand. J. Urol. Nephrol..

[82] Himmelfarb J., Stenvinkel P., Ikizler T.A., Hakim R.M. (2002). The elephant in uremia: Oxidant stress as a unifying concept of cardiovascular disease in uremia. Kidney Int..

[83] Locatelli F., Canaud B., Eckardt K.-U., Stenvinkel P., Wanner C., Zoccali C. (2003). Oxidative stress in end-stage renal disease: An emerging threat to patient outcome. Nephrol. Dial. Transplant..

[84] Ross R. (1999). Atherosclerosis—An inflammatory disease. N. Engl. J. Med..

[85] Habermann E. (1991). Problems in long-term therapy from the viewpoint of the pharmacologist. Verh. Dtsch. Ges. Inn. Med..

[86] Wolf M. (2009). Fibroblast growth factor 23 and the future of phosphorus management. Curr. Opin. Nephrol. Hypertens..

[87] Moe S.M., Reslerova M., Ketteler M., O’Neill K., Duan D., Koczman J., Westenfeld R., Jahnen-Dechent W., Chen N.X. (2005). Role of calcification inhibitors in the pathogenesis of vascular calcification in chronic kidney disease (CKD). Kidney Int..

[88] Schinke T., Karsenty G. (2000). Vascular calcification--a passive process in need of inhibitors. Nephrol. Dial. Transplant..

[89] Balci M., Kirkpantur A., Gulbay M., Gurbuz O.A. (2010). Plasma fibroblast growth factor-23 levels are independently associated with carotid artery atherosclerosis in maintenance hemodialysis patients. Hemodial. Int..

[90] Desjardins L., Liabeuf S., Renard C., Lenglet A., Lemke H.D., Choukroun G., Drueke T.B., Massy Z.A., European Uremic Toxin Work G. (2012). FGF23 is independently associated with vascular calcification but not bone mineral density in patients at various CKD stages. Osteoporos. Int..

[91] Coppolino G., Bolignano D., Gareri P., Ruberto C., Andreucci M., Ruotolo G., Rocca M., Castagna A. (2018). Kidney function and cognitive decline in frail elderly: Two faces of the same coin?. Int. Urol. Nephrol..

[92] Bolignano D., Greco M., Arcidiacono V., Tripolino O., Vita C., Provenzano M., Donato C., Chiarella S., Fuiano G., De Sarro G. (2021). Increased circulating Cathepsin-K levels reflect PTH control in chronic hemodialysis patients. J. Nephrol..

[93] Bolignano D., Greco M., Arcidiacono V., Tripolino O., Vita C., Provenzano M., Donato C., Chiarella S., Fuiano G., De Sarro G. (2021). Cathepsin-K is a potential cardiovascular risk biomarker in prevalent hemodialysis patients. Int. Urol. Nephrol..

[94] Bolignano D., Greco M., Arcidiacono V., Presta P., Caglioti A., Russo E., Andreucci M., Tripolino O., Carullo N., Foti D.P. (2022). Decreased Cathepsin-K Mirrors the Severity of Subclinical Atherosclerosis in Kidney Transplant Recipients. Rev. Cardiovasc. Med..

[95] Vassalle C., Mazzone A. (2016). Bone loss and vascular calcification: A bi-directional interplay?. Vasc. Pharmacol..

[96] Saftig P., Hunziker E., Everts V., Jones S., Boyde A., Wehmeyer O., Suter A., von Figura K. (2000). Functions of cathepsin K in bone resorption. Lessons from cathepsin K deficient mice. Adv. Exp. Med. Biol..

[97] Feenstra L., Reijrink M., Pasch A., Smith E.R., Visser L.M., Bulthuis M., Lodewijk M.E., Mastik M.F., Greuter M.J.W., Slart R.H.J.A. (2024). Calciprotein particle counts associate with vascular remodelling in chronic kidney disease. Cardiovasc. Res..

[98] Lian Y., Li Y., Liu A., Ghosh S., Shi Y., Huang H. (2023). Dietary antioxidants and vascular calcification: From pharmacological mechanisms to challenges. Biomed. Pharmacother..

[99] Al-Qaridhi A., Ghosh S., Luo D., Huang H. (2022). Magnesium and Zinc Intake Ratio Mediates the Increase of Coronary Artery Calcification through Upregulating Interleukin 6. Libyan J. Med..

[100] Moe S.M., Chen N.X. (2008). Mechanisms of vascular calcification in chronic kidney disease. J. Am. Soc. Nephrol..

[101] Dube P., DeRiso A., Patel M., Battepati D., Khatib-Shahidi B., Sharma H., Gupta R., Malhotra D., Dworkin L., Haller S. (2021). Vascular Calcification in Chronic Kidney Disease: Diversity in the Vessel Wall. Biomedicines.

[102] Radloff J., Latic N., Pfeiffenberger U., Schuler C., Tangermann S., Kenner L., Erben R.G. (2021). A phosphate and calcium-enriched diet promotes progression of 5/6-nephrectomy-induced chronic kidney disease in C57BL/6 mice. Sci. Rep..

[103] Evenepoel P., Wolf M. (2013). A balanced view of calcium and phosphate homeostasis in chronic kidney disease. Kidney Int..

[104] Voelkl J., Lang F., Eckardt K.U., Amann K., Kuro O.M., Pasch A., Pieske B., Alesutan I. (2019). Signaling pathways involved in vascular smooth muscle cell calcification during hyperphosphatemia. Cell Mol. Life Sci..

[105] Bourne L.E., Wheeler-Jones C.P., Orriss I.R. (2021). Regulation of mineralisation in bone and vascular tissue: A comparative review. J. Endocrinol..

[106] Nakano-Kurimoto R., Ikeda K., Uraoka M., Nakagawa Y., Yutaka K., Koide M., Takahashi T., Matoba S., Yamada H., Okigaki M. (2009). Replicative senescence of vascular smooth muscle cells enhances the calcification through initiating the osteoblastic transition. Am. J. Physiol. Heart Circ. Physiol..

[107] Bundy K., Boone J., Simpson C.L. (2021). Wnt Signaling in Vascular Calcification. Front. Cardiovasc. Med..

[108] Viegas C., Araújo N., Marreiros C., Simes D. (2019). The interplay between mineral metabolism, vascular calcification and inflammation in Chronic Kidney Disease (CKD): Challenging old concepts with new facts. Aging.

[109] Hénaut L., Sanchez-Nino M.D., Aldamiz-Echevarría Castillo G., Sanz A.B., Ortiz A. (2016). Targeting local vascular and systemic consequences of inflammation on vascular and cardiac valve calcification. Expert. Opin. Ther. Targets.

[110] Hu C.-T., Shao Y.-D., Liu Y.-Z., Xiao X., Cheng Z.-B., Qu S.-L., Huang L., Zhang C. (2021). Oxidative stress in vascular calcification. Clin. Chim. Acta.

[111] Liu S.M., Zhang Y.R., Chen Y., Ji D.R., Zhao J., Fu S., Jia M.Z., Yu Y.R., Tang C.S., Huang W. (2022). Intermedin Alleviates Vascular Calcification in CKD through Sirtuin 3-Mediated Inhibition of Mitochondrial Oxidative Stress. Pharmaceuticals.

[112] Yamada S., Giachelli C.M. (2017). Vascular calcification in CKD-MBD: Roles for phosphate, FGF23, and Klotho. Bone.

[113] Yan J., Wang J., He J.C., Zhong Y. (2022). Sirtuin 1 in Chronic Kidney Disease and Therapeutic Potential of Targeting Sirtuin 1. Front. Endocrinol..

[114] Gao D., Zuo Z., Tian J., Ali Q., Lin Y., Lei H., Sun Z. (2016). Activation of SIRT1 Attenuates Klotho Deficiency-Induced Arterial Stiffness and Hypertension by Enhancing AMP-Activated Protein Kinase Activity. Hypertension.

[115] Bao W.-H., Yang W.-L., Su C.-Y., Lu X.-H., He L., Zhang A.-H. (2023). Relationship between gut microbiota and vascular calcification in hemodialysis patients. Ren. Fail..

[116] Barrett H., O’Keeffe M., Kavanagh E., Walsh M., O’Connor E.M. (2018). Is Matrix Gla Protein Associated with Vascular Calcification? A Systematic Review. Nutrients.

[117] Shioi A., Morioka T., Shoji T., Emoto M. (2020). The Inhibitory Roles of Vitamin K in Progression of Vascular Calcification. Nutrients.

[118] Bellone F., Cinquegrani M., Nicotera R., Carullo N., Casarella A., Presta P., Andreucci M., Squadrito G., Mandraffino G., Prunesti M. (2022). Role of Vitamin K in Chronic Kidney Disease: A Focus on Bone and Cardiovascular Health. Int. J. Mol. Sci..

[119] Westenfeld R., Jahnen-Dechent W., Ketteler M. (2007). Vascular calcification and fetuin-A deficiency in chronic kidney disease. Trends Cardiovasc. Med..

[120] Westenfeld R., Schafer C., Smeets R., Brandenburg V.M., Floege J., Ketteler M., Jahnen-Dechent W. (2007). Fetuin-A (AHSG) prevents extraosseous calcification induced by uraemia and phosphate challenge in mice. Nephrol. Dial. Transplant..

[121] Musolino M., Greco M., D’Agostino M., Tripodi L., Misiti R., Dragone F., Cianfrone P., Zicarelli M., Foti D.P., Andreucci M. (2024). Urinary Post-Translationally Modified Fetuin-A (uPTM-FetA) in Chronic Kidney Disease Patients with and without Diabetic Kidney Disease. Medicina.

[122] Makarović S., Makarović Z., Steiner R., Mihaljević I., Milas-Ahić J. (2015). Osteoprotegerin and Vascular Calcification: Clinical and Prognostic Relevance. Coll. Antropol..

[123] Matsushita K., Coresh J., Sang Y., Chalmers J., Fox C., Guallar E., Jafar T., Jassal S.K., Landman G.W.D., Muntner P. (2015). Estimated glomerular filtration rate and albuminuria for prediction of cardiovascular outcomes: A collaborative meta-analysis of individual participant data. Lancet Diabetes Endocrinol..

[124] London G.M., Guerin A.P., Marchais S.J., Metivier F., Pannier B., Adda H. (2003). Arterial media calcification in end-stage renal disease: Impact on all-cause and cardiovascular mortality. Nephrol. Dial. Transplant..

[125] Russo D., Corrao S., Battaglia Y., Andreucci M., Caiazza A., Carlomagno A., Lamberti M., Pezone N., Pota A., Russo L. (2011). Progression of coronary artery calcification and cardiac events in patients with chronic renal disease not receiving dialysis. Kidney Int..

[126] Russo D., Morrone L.F., Errichiello C., De Gregorio M.G., Imbriaco M., Battaglia Y., Russo L., Andreucci M., Di Iorio B.R. (2014). Impact of BMI on cardiovascular events, renal function, and coronary artery calcification. Blood Purif..

[127] Schwarz U., Buzello M., Ritz E., Stein G., Raabe G., Wiest G., Mall G., Amann K. (2000). Morphology of coronary atherosclerotic lesions in patients with end-stage renal failure. Nephrol. Dial. Transplant..

[128] Nakamura S., Ishibashi-Ueda H., Niizuma S., Yoshihara F., Horio T., Kawano Y. (2009). Coronary Calcification in Patients with Chronic Kidney Disease and Coronary Artery Disease. Clin. J. Am. Soc. Nephrol..

[129] Piers L.H., Touw H.R.W., Gansevoort R., Franssen C.F.M., Oudkerk M., Zijlstra F., Tio R.A. (2009). Relation of aortic valve and coronary artery calcium in patients with chronic kidney disease to the stage and etiology of the renal disease. Am. J. Cardiol..

[130] Tomiyama C., Carvalho A.B., Higa A., Jorgetti V., Draibe S.A., Canziani M.E.F. (2010). Coronary calcification is associated with lower bone formation rate in CKD patients not yet in dialysis treatment. J. Bone Miner. Res..

[131] Aleksova J., Kurniawan S., Vucak-Dzumhur M., Kerr P., Ebeling P.R., Milat F., Elder G.J. (2018). Aortic vascular calcification is inversely associated with the trabecular bone score in patients receiving dialysis. Bone.

[132] Chen T.-Y., Yang J., Zuo L., Wang L., Wang L.-F. (2022). Relationship of abdominal aortic calcification with lumbar vertebral volumetric bone mineral density assessed by quantitative computed tomography in maintenance hemodialysis patients. Arch. Osteoporos..

[133] Kraus M.A., Kalra P.A., Hunter J., Menoyo J., Stankus N. (2015). The prevalence of vascular calcification in patients with end-stage renal disease on hemodialysis: A cross-sectional observational study. Ther. Adv. Chronic Dis..

[134] Pencak P., Czerwieńska B., Ficek R., Wyskida K., Kujawa-Szewieczek A., Olszanecka-Glinianowicz M., Więcek A., Chudek J. (2013). Calcification of coronary arteries and abdominal aorta in relation to traditional and novel risk factors of atherosclerosis in hemodialysis patients. BMC Nephrol..

[135] Toussaint N.D., Lau K.K., Strauss B.J., Polkinghorne K.R., Kerr P.G. (2008). Associations between vascular calcification, arterial stiffness and bone mineral density in chronic kidney disease. Nephrol. Dial. Transplant..

[136] Narula N., Dannenberg A.J., Olin J.W., Bhatt D.L., Johnson K.W., Nadkarni G., Min J., Torii S., Poojary P., Anand S.S. (2018). Pathology of Peripheral Artery Disease in Patients With Critical Limb Ischemia. J. Am. Coll. Cardiol..

[137] O’Neill W.C., Han K.H., Schneider T.M., Hennigar R.A. (2015). Prevalence of nonatheromatous lesions in peripheral arterial disease. Arterioscler. Thromb. Vasc. Biol..

[138] Roijers J.P., Rakké Y.S., Hopmans C.J., Buimer M.G., Ho G.H., de Groot H.G.W., Veen E.J., Mulder P.G.H., van der Laan L. (2020). A mortality prediction model for elderly patients with critical limb ischemia. J. Vasc. Surg..

[139] Damjanovic T., Djuric S., Schlieper G., Markovic N., Dimkovic S., Radojicic Z., Krüger T., Floege J., Dimkovic N. (2009). Clinical features of hemodialysis patients with intimal versus medial vascular calcifications. J. Nephrol..

[140] Gelev S., Spasovski G., Trajkovski Z., Damjanovski G., Amitov V., Selim G., Dzekova P., Sikole A. (2008). Factors associated with various arterial calcifications in haemodialysis patients. Prilozi.

[141] Libby P. (2013). Collagenases and cracks in the plaque. J. Clin. Investig..

[142] Mori H., Torii S., Kutyna M., Sakamoto A., Finn A.V., Virmani R. (2018). Coronary Artery Calcification and its Progression: What Does it Really Mean?. JACC Cardiovasc. Imaging.

[143] Nakahara T., Dweck M.R., Narula N., Pisapia D., Narula J., Strauss H.W. (2017). Coronary Artery Calcification: From Mechanism to Molecular Imaging. JACC Cardiovasc. Imaging.

[144] Kelly-Arnold A., Maldonado N., Laudier D., Aikawa E., Cardoso L., Weinbaum S. (2013). Revised microcalcification hypothesis for fibrous cap rupture in human coronary arteries. Proc. Natl. Acad. Sci. USA.

[145] Criqui M.H., Denenberg J.O., Ix J.H., McClelland R.L., Wassel C.L., Rifkin D.E., Carr J.J., Budoff M.J., Allison M.A. (2014). Calcium density of coronary artery plaque and risk of incident cardiovascular events. JAMA.

[146] Shreya D., Zamora D.I., Patel G.S., Grossmann I., Rodriguez K., Soni M., Joshi P.K., Patel S.C., Sange I. (2021). Coronary Artery Calcium Score—A Reliable Indicator of Coronary Artery Disease?. Cureus.

[147] Lehmann N., Erbel R., Mahabadi A.A., Rauwolf M., Mohlenkamp S., Moebus S., Kalsch H., Budde T., Schmermund A., Stang A. (2018). Value of Progression of Coronary Artery Calcification for Risk Prediction of Coronary and Cardiovascular Events: Result of the HNR Study (Heinz Nixdorf Recall). Circulation.

[148] Ando G., Vizzari G., Niccoli G., Calabro P., Zimarino M., Spaccarotella C., De Rosa S., Piccolo R., Gragnano F., Mancone M. (2021). Evaluation and percutaneous treatment of severely calcified coronary lesions. G. Ital. Cardiol..

[149] Panuccio G., Werner G.S., De Rosa S., Torella D., Leistner D.M., Siegrist P.T., Haghikia A., Skurk C., Mashayekhi K., Landmesser U. (2024). Full-Moon Coronary Calcification as Detected With Computed Tomography Angiography in Chronic Total Occlusion Percutaneous Coronary Intervention. Am. J. Cardiol..

[150] Zaffino P., Spadea M.F., Indolfi C., De Rosa S. (2022). CoroFinder: A New Tool for Real Time Detection and Tracking of Coronary Arteries in Contrast-Free Cine-Angiography. J. Pers. Med..

[151] Pandya V., Rao A., Chaudhary K. (2015). Lipid abnormalities in kidney disease and management strategies. WJN.

[152] Hou W., Lv J., Perkovic V., Yang L., Zhao N., Jardine M.J., Cass A., Zhang H., Wang H. (2013). Effect of statin therapy on cardiovascular and renal outcomes in patients with chronic kidney disease: A systematic review and meta-analysis. Eur. Heart J..

[153] Wanner C., Krane V., März W., Olschewski M., Mann J.F.E., Ruf G., Ritz E., German D., Dialysis Study I. (2005). Atorvastatin in patients with type 2 diabetes mellitus undergoing hemodialysis. N. Engl. J. Med..

[154] Fellström B.C., Jardine A.G., Schmieder R.E., Holdaas H., Bannister K., Beutler J., Chae D.-W., Chevaile A., Cobbe S.M., Grönhagen-Riska C. (2009). Rosuvastatin and cardiovascular events in patients undergoing hemodialysis. N. Engl. J. Med..

[155] Ceneri N., Zhao L., Young B.D., Healy A., Coskun S., Vasavada H., Yarovinsky T.O., Ike K., Pardi R., Qin L. (2017). Rac2 Modulates Atherosclerotic Calcification by Regulating Macrophage Interleukin-1β Production. Arterioscler. Thromb. Vasc. Biol..

[156] Healy A., Berus J.M., Christensen J.L., Lee C., Mantsounga C., Dong W., Watts J.P., Assali M., Ceneri N., Nilson R. (2020). Statins Disrupt Macrophage Rac1 Regulation Leading to Increased Atherosclerotic Plaque Calcification. Arterioscler. Thromb. Vasc. Biol..

[157] Puri R., Nicholls S.J., Shao M., Kataoka Y., Uno K., Kapadia S.R., Tuzcu E.M., Nissen S.E. (2015). Impact of statins on serial coronary calcification during atheroma progression and regression. J. Am. Coll. Cardiol..

[158] Himmelfarb J., Ikizler T.A. (2010). Hemodialysis. N. Engl. J. Med..

[159] Kwan B.C., Kronenberg F., Beddhu S., Cheung A.K. (2007). Lipoprotein metabolism and lipid management in chronic kidney disease. J. Am. Soc. Nephrol..

[160] Rader F., Sachdev E., Arsanjani R., Siegel R.J. (2015). Left ventricular hypertrophy in valvular aortic stenosis: Mechanisms and clinical implications. Am. J. Med..

[161] Churchill T.W., Yucel E., Deferm S., Levine R.A., Hung J., Bertrand P.B. (2022). Mitral Valve Dysfunction in Patients With Annular Calcification: JACC Review Topic of the Week. J. Am. Coll. Cardiol..

[162] Sabatino J., Wicik Z., De Rosa S., Eyileten C., Jakubik D., Spaccarotella C., Mongiardo A., Postula M., Indolfi C. (2019). MicroRNAs fingerprint of bicuspid aortic valve. J. Mol. Cell Cardiol..

[163] Luraghi G., Matas J.F.R., Beretta M., Chiozzi N., Iannetti L., Migliavacca F. (2021). The impact of calcification patterns in transcatheter aortic valve performance: A fluid-structure interaction analysis. Comput. Methods Biomech. Biomed. Eng..

[164] Hill K.M., Martin B.R., Wastney M.E., McCabe G.P., Moe S.M., Weaver C.M., Peacock M. (2013). Oral calcium carbonate affects calcium but not phosphorus balance in stage 3-4 chronic kidney disease. Kidney Int..

[165] Pisano A., D’Arrigo G., Coppolino G., Bolignano D. (2018). Biotic Supplements for Renal Patients: A Systematic Review and Meta-Analysis. Nutrients.

[166] Tsai P.-H., Chung C.-H., Chien W.-C., Chu P. (2020). Effects of calcium-containing phosphate binders on cardiovascular events and mortality in predialysis CKD stage 5 patients. PLoS ONE.

[167] Briese S., Wiesner S., Will J.C., Lembcke A., Opgen-Rhein B., Nissel R., Wernecke K.D., Andreae J., Haffner D., Querfeld U. (2006). Arterial and cardiac disease in young adults with childhood-onset end-stage renal disease-impact of calcium and vitamin D therapy. Nephrol. Dial. Transplant..

[168] McCabe K.M., Zelt J.G., Kaufmann M., Laverty K., Ward E., Barron H., Jones G., Adams M.A., Holden R.M. (2018). Calcitriol Accelerates Vascular Calcification Irrespective of Vitamin K Status in a Rat Model of Chronic Kidney Disease with Hyperphosphatemia and Secondary Hyperparathyroidism. J. Pharmacol. Exp. Ther..

[169] Hénaut L., Boudot C., Massy Z.A., Lopez-Fernandez I., Dupont S., Mary A., Drüeke T.B., Kamel S., Brazier M., Mentaverri R. (2014). Calcimimetics increase CaSR expression and reduce mineralization in vascular smooth muscle cells: Mechanisms of action. Cardiovasc. Res..

[170] Addi T., Dou L., Burtey S. (2018). Tryptophan-Derived Uremic Toxins and Thrombosis in Chronic Kidney Disease. Toxins.

[171] Rabelink T.J., Zwaginga J.J., Koomans H.A., Sixma J.J. (1994). Thrombosis and hemostasis in renal disease. Kidney Int..

[172] Janssen M.J., van der Meulen J. (1996). The bleeding risk in chronic haemodialysis: Preventive strategies in high-risk patients. Neth. J. Med..

[173] Baylis C. (2008). Nitric oxide deficiency in chronic kidney disease. Am. J. Physiol. Ren. Physiol..

[174] Chen J.Y., Ye Z.X., Wang X.F., Chang J., Yang M.W., Zhong H.H., Hong F.F., Yang S.L. (2018). Nitric oxide bioavailability dysfunction involves in atherosclerosis. Biomed. Pharmacother..

[175] Glorieux G., Cohen G., Jankowski J., Vanholder R. (2009). Platelet/Leukocyte activation, inflammation, and uremia. Semin. Dial..

[176] Schurgers L.J., Akbulut A.C., Kaczor D.M., Halder M., Koenen R.R., Kramann R. (2018). Initiation and Propagation of Vascular Calcification Is Regulated by a Concert of Platelet- and Smooth Muscle Cell-Derived Extracellular Vesicles. Front. Cardiovasc. Med..

[177] Foresta C., Strapazzon G., De Toni L., Fabris F., Grego F., Gerosa G., Vettore S., Garolla A. (2013). Platelets express and release osteocalcin and co-localize in human calcified atherosclerotic plaques. J. Thromb. Haemost..

[178] Mizokami A., Kawakubo-Yasukochi T., Hirata M. (2017). Osteocalcin and its endocrine functions. Biochem. Pharmacol..

[179] Gong S., Wang C., Xiong J., Zhao J., Yang K. (2022). Activated Platelets, the Booster of Chronic Kidney Disease and Cardiovascular Complications. Kidney Dis..

[180] Cernaro V., Coppolino G., Visconti L., Rivoli L., Lacquaniti A., Santoro D., Buemi A., Loddo S., Buemi M. (2019). Erythropoiesis and chronic kidney disease-related anemia: From physiology to new therapeutic advancements. Med. Res. Rev..

[181] Buemi M., Senatore M., Gallo G.C., Crasci E., Campo S., Sturiale A., Coppolino G., Bolignano D., Frisina N. (2007). Pulmonary hypertension and erythropoietin. Kidney Blood Press. Res..

[182] Sage A.P., Tintut Y., Demer L.L. (2010). Regulatory mechanisms in vascular calcification. Nat. Rev. Cardiol..

[183] Bernelot Moens S.J., Verweij S.L., van der Valk F.M., van Capelleveen J.C., Kroon J., Versloot M., Verberne H.J., Marquering H.A., Duivenvoorden R., Vogt L. (2017). Arterial and Cellular Inflammation in Patients with CKD. J. Am. Soc. Nephrol..

[184] Shanahan C.M. (2013). Mechanisms of vascular calcification in CKD-evidence for premature ageing?. Nat. Rev. Nephrol..

[185] Foley R.N., Parfrey P.S., Sarnak M.J. (1998). Clinical epidemiology of cardiovascular disease in chronic renal disease. Am. J. Kidney Dis. Off. J. Natl. Kidney Found..

[186] Nelson A.J., Worthley S.G., Cameron J.D., Willoughby S.R., Piantadosi C., Carbone A., Dundon B.K., Leung M.C., Hope S.A., Meredith I.T. (2009). Cardiovascular magnetic resonance-derived aortic distensibility: Validation and observed regional differences in the elderly. J. Hypertens..

[187] Braun J., Oldendorf M., Moshage W., Heidler R., Zeitler E., Luft F.C. (1996). Electron beam computed tomography in the evaluation of cardiac calcification in chronic dialysis patients. Am. J. Kidney Dis. Off. J. Natl. Kidney Found..

[188] Budoff M.J., Rader D.J., Reilly M.P., Mohler E.R., Lash J., Yang W., Rosen L., Glenn M., Teal V., Feldman H.I. (2011). Relationship of estimated GFR and coronary artery calcification in the CRIC (Chronic Renal Insufficiency Cohort) Study. Am. J. Kidney Dis. Off. J. Natl. Kidney Found..

[189] Chen J., Budoff M.J., Reilly M.P., Yang W., Rosas S.E., Rahman M., Zhang X., Roy J.A., Lustigova E., Nessel L. (2017). Coronary Artery Calcification and Risk of Cardiovascular Disease and Death Among Patients With Chronic Kidney Disease. JAMA Cardiol..

[190] Marwick T.H., Amann K., Bangalore S., Cavalcante J.L., Charytan D.M., Craig J.C., Gill J.S., Hlatky M.A., Jardine A.G., Landmesser U. (2019). Chronic kidney disease and valvular heart disease: Conclusions from a Kidney Disease: Improving Global Outcomes (KDIGO) Controversies Conference. Kidney Int..

[191] Wang Z., Jiang A., Wei F., Chen H. (2018). Cardiac valve calcification and risk of cardiovascular or all-cause mortality in dialysis patients: A meta-analysis. BMC Cardiovasc. Disord..

[192] Niu Q., Hong Y., Lee C.-H., Men C., Zhao H., Zuo L. (2018). Abdominal aortic calcification can predict all-cause mortality and CV events in dialysis patients: A systematic review and meta-analysis. PLoS ONE.

[193] Manzoor S., Ahmed S., Ali A., Han K.H., Sechopoulos I., O’Neill A., Fei B., O’Neill W.C. (2018). Progression of Medial Arterial Calcification in CKD. Kidney Int. Rep..

[194] Abou-Hassan N., Tantisattamo E., D’Orsi E.T., O’Neill W.C. (2015). The clinical significance of medial arterial calcification in end-stage renal disease in women. Kidney Int..

[195] Disthabanchong S., Boongird S. (2017). Role of different imaging modalities of vascular calcification in predicting outcomes in chronic kidney disease. World J. Nephrol..

[196] Zaimi M., Grapsa E. (2024). Current therapeutic approach of chronic kidney disease-mineral and bone disorder. Ther. Apher. Dial..

[197] Brown E.M., Pollak M., Hebert S.C. (1995). Sensing of extracellular Ca2+ by parathyroid and kidney cells: Cloning and characterization of an extracellular Ca2+-sensing receptor. Am. J. Kidney Dis..

[198] Rodríguez-Ortiz M.E., Pendón-Ruiz De Mier M.V., Rodríguez M. (2019). Parathyroidectomy in dialysis patients: Indications, methods, and consequences. Semin. Dial..

[199] Hu L., Napoletano A., Provenzano M., Garofalo C., Bini C., Comai G., La Manna G. (2022). Mineral Bone Disorders in Kidney Disease Patients: The Ever-Current Topic. Int. J. Mol. Sci..

[200] Sprague S.M., Martin K.J., Coyne D.W. (2021). Phosphate Balance and CKD-Mineral Bone Disease. Kidney Int. Rep..

[201] Bover J., Bailone L., López-Báez V., Benito S., Ciceri P., Galassi A., Cozzolino M. (2017). Osteoporosis, bone mineral density and CKD-MBD: Treatment considerations. J. Nephrol..

[202] Cupisti A., Gallieni M., Rizzo M.A., Caria S., Meola M., Bolasco P. (2013). Phosphate control in dialysis. Int. J. Nephrol. Renov. Dis..

